# Supplementary material for: The Role of the Chronotype in Developing an Excessive Body Weight and Its Complications—A Narrative Review
Source: Nutrients. 2024 Dec 28;17(1):80. doi: 10.3390/nu17010080 (PMC11723089; doi:10.3390/nu17010080)
Supplement: Supplementary file 1 [file nutrients-17-00080-s001.zip › nutrients-3398129-supplementary.pdf]

Searching strategy

A literature search was conducted via the electronic databases PubMed, Medline, and Web of Science between August and November 2024. No restriction was set on the year of publication; nonetheless, studies were limited to those published in English, full text, and conducted among humans only. Various populations were analyzed (adults and children), while pregnant and lactating women were excluded. Two reviewers (M.P. and M.M) completed the data searching.

The search terms used for this narrative review were:
1. Chronotype OR circadian rhythm OR morningness OR eveningness;
2. Obesity OR overweight OR eating behavior OR physical activity OR hypertension OR metabolic syndrome OR diabetes OR sleep apnea OR liver diseases;
3. 1 AND 2.
